# Effectiveness of Current Diagnostic Methods in the Early Detection of Diabetic Peripheral Neuropathy: Are We Missing the Window?

**DOI:** 10.7759/cureus.106429

**Published:** 2026-04-04

**Authors:** Amara Liaqat

**Affiliations:** 1 Internal Medicine, Aberdeen Royal Infirmary, NHS Grampian, Aberdeen, GBR

**Keywords:** corneal confocal microscopy, diabetic foot, diabetic peripheral neuropathy, early diagnosis, skin biopsy, small-fiber neuropathy

## Abstract

Diabetic peripheral neuropathy (DPN) is one of the most common long-term complications of diabetes and a major cause of foot ulceration, pain, balance impairment, and lower-limb amputation. Nerve injury may begin early, but many patients remain undiagnosed until they already have clinically important sensory loss. A major reason is that early neuropathic change may involve small sensory and autonomic fibers, whereas routine clinic screening is largely designed to identify established large-fiber dysfunction and loss of protective sensation. This narrative review evaluates the strengths and limitations of current diagnostic methods for DPN and considers whether routine practice misses the optimal window for early detection. For transparency, the literature was narratively reviewed using PubMed/MEDLINE (Medical Literature Analysis and Retrieval System Online), Scopus, and Google Scholar, focusing mainly on English-language studies, guidelines, landmark trials, and review articles published between 2000 and March 2026, while also including older seminal papers where relevant. Search terms covered DPN, diabetic sensorimotor polyneuropathy, small-fiber neuropathy, corneal confocal microscopy, skin biopsy, quantitative sensory testing, sudomotor testing, point-of-care nerve conduction, neurofilament light chain, prediabetes, metabolic syndrome, screening, diagnosis, and guidelines. We reviewed bedside screening tools, structured clinical scores, nerve conduction studies, and newer approaches that assess small-fiber damage, including skin biopsy, corneal confocal microscopy, quantitative sensory testing, and sudomotor assessment. We also discuss point-of-care nerve conduction devices, autonomic testing, emerging blood biomarkers, and wearable technologies. Traditional bedside tools such as the 10 g monofilament and vibration testing remain essential for ulcer-risk assessment, but their sensitivity for early disease is limited. Nerve conduction studies are useful for confirming large-fiber neuropathy and excluding alternative diagnoses, yet they may remain normal in early or predominantly small-fiber disease. Methods that focus on small-fiber pathology have shown promise for earlier detection, but broader implementation is restricted by cost, access, training requirements, variable standardization, and limited outcome data showing that earlier detection consistently changes long-term endpoints. No single test is ideal. In current practice, the most realistic strategy is a risk-based, multimodal approach: continue universal annual screening for all people with diabetes, distinguish clearly between screening for ulcer risk and diagnosing neuropathy, take symptoms seriously even when routine tests are normal, and refer selected symptomatic or high-risk patients for further assessment where small-fiber testing is available.

## Introduction and background

Diabetic peripheral neuropathy (DPN) is one of the most frequent chronic complications of diabetes mellitus. It is closely linked to foot ulceration, falls, neuropathic pain, reduced quality of life, and amputation [1,2]. Despite this, early disease is often missed in routine practice. Many patients are diagnosed only when numbness is obvious, protective sensation has already been lost, or foot risk has clearly increased.

The explanation is not simply underscreening, but also a mismatch between the biology of early disease and the tools most often used in the clinic. Small unmyelinated C fibers and thinly myelinated A-delta fibers may be affected early in at least a substantial subset of patients, particularly those with painful or metabolic neuropathy phenotypes [2-5]. These fibers mediate pain, temperature, and autonomic function. By contrast, the tools most commonly used in routine diabetic foot screening, such as monofilament and tuning-fork testing, mainly detect more established large-fiber dysfunction and loss of protective sensation. A patient may therefore have clinically meaningful neuropathic injury while standard bedside screening is still normal.

It is also important to avoid treating DPN as completely uniform across type 1 and type 2 diabetes. Hyperglycemia is central in both, but obesity, dyslipidemia, insulin resistance, and chronic inflammation appear especially important in type 2 diabetes and in neuropathy associated with prediabetes or metabolic syndrome [4-7]. Large trials support the view that intensive glycemic control has a stronger preventive effect on neuropathy in type 1 diabetes than in type 2 diabetes [6,7]. This difference matters when discussing the value of early detection and the practical actions that should follow it.

The aim of this review is therefore more specific than simply listing available tests. It examines whether current diagnostic pathways separate three related but distinct clinical purposes clearly enough: screening for foot risk, diagnosing neuropathy, and phenotyping early or predominantly small-fiber disease. This distinction is essential because a tool can be highly useful for ulcer prevention without being sensitive for the earliest neuropathic change. If neuropathy is recognized earlier, clinicians may have a better opportunity to intensify glycemic and cardiovascular risk-factor control, review potentially neurotoxic contributors, address painful symptoms promptly, and identify patients who need more detailed neurophysiological or small-fiber assessment.

Methods

This is a narrative review. The literature was identified through PubMed/MEDLINE (Medical Literature Analysis and Retrieval System Online), Scopus, and Google Scholar up to March 2026, focusing mainly on studies since 2000, with earlier key references included where needed. Priority was given to guidelines, consensus statements, validation studies, and representative reviews relevant to early detection of DPN in real-world practice.

## Review

​​​​​​Why early DPN is frequently overlooked

Early DPN is easy to miss for several reasons. Symptoms may be absent, mild, intermittent, or difficult for patients to describe. Patients may report burning, tingling, pins and needles, altered temperature sensation, or gait insecurity rather than classic neuropathic pain. In routine diabetes care, screening often prioritises loss of protective sensation because of its direct link to ulcer risk, which is clinically important but does not fully address early neuropathic change. In addition, early neuropathy may predominantly involve small sensory fibres, whereas commonly used bedside tools are more sensitive to established large-fibre dysfunction. As a result, clinically meaningful neuropathy may still be present despite normal routine screening tests [2,4,5,8].

Another issue is diagnostic overlap. Vitamin B12 deficiency, alcohol use, hypothyroidism, chronic kidney disease, chemotherapy, spinal disease, entrapment neuropathies, and other neurological disorders may mimic or coexist with DPN [2,9]. Because of this, clinicians may hesitate to label early symptoms as neuropathy when routine examination is normal. Delay is particularly likely when symptoms are sensory, distal, and fluctuating, but reflexes and monofilament testing remain preserved.

Current annual screening recommendations are necessary, but annual screening does not automatically equal early diagnosis. A patient may attend regular diabetes review, have normal monofilament testing for years, and still be developing painful or autonomic small-fiber injury. Studies comparing simple clinic tests against electrophysiological or more specialized measures have shown that routine bedside tools are useful but less sensitive for early disease than for more advanced neuropathy [2,8]. In practical terms, current screening methodologies may miss neuropathy in patients whose symptoms, risk profile, or small-fiber pathology precede overt large-fiber abnormalities.

Guideline context: what current screening does and does not do

Major diabetes guidelines recommend annual assessment for neuropathy and foot risk using history, examination, and simple bedside tests such as monofilament with another modality, for example, vibration, pinprick, or ankle reflexes [1,10-12]. These recommendations are suitable for population screening and ulcer prevention, but they do not fully address early small-fiber neuropathy. The main gap is in patients with neuropathic symptoms or autonomic complaints whose bedside tests and even conventional nerve conduction studies remain normal. The issue is therefore not whether annual screening should continue, but when assessment should be escalated despite negative first-line tests.

Screening, diagnosis, and phenotyping: a useful clinical distinction

Three related but distinct clinical tasks should be separated. Screening identifies patients at risk of foot injury or established neuropathy, diagnosis confirms neuropathy and considers alternative causes, and phenotyping defines whether the disease is predominantly small-fibre, large-fibre, painful, autonomic, or atypical, which helps guide further evaluation and management. Problems arise when a single bedside tool is expected to fulfil all three roles. The monofilament is useful for detecting loss of protective sensation and stratifying ulcer risk, but it is not sensitive for early small-fibre disease. Similarly, conventional nerve conduction studies are valuable for confirming large-fibre neuropathy and excluding alternative diagnoses, but they may remain normal in early or small-fibre-predominant disease [2,8,9,13,14].

Bedside Screening Tools

The 10 g monofilament and 128 Hz tuning fork remain central to routine diabetic foot assessment [9-12]. They are cheap, quick, and easy to use. Their major strength is practical risk stratification. When monofilament sensation is lost, the patient is already at increased risk of ulceration and requires closer surveillance, education, and footwear advice. However, these tools are not very good at identifying neuropathy initially. Their performance is strongest for established sensory loss rather than early small-fiber injury. They should therefore be retained as front-line screening tests, but a normal result should not be interpreted as proof that early neuropathy is absent.

Clinical Scoring Systems

Structured clinical scores help make assessments more consistent. The Michigan Neuropathy Screening Instrument and the Toronto Clinical Scoring System are among the best-known approaches [15-17]. They combine symptoms, reflexes, and sensory findings into a more organized bedside evaluation and are useful for documentation and longitudinal follow-up. In addition, the Utah Early Neuropathy Scale focuses more specifically on early sensory-predominant neuropathy and small-fiber-predominant findings, making it relevant when clinicians want a bedside framework that is more sensitive to subtle early abnormalities [18]. Even so, these scales still depend partly on abnormalities that may emerge after the earliest nerve injury has started, so they improve assessment but do not eliminate the need for escalation in selected patients.

Nerve Conduction Studies (NCS)

NCSs remain highly valuable when large-fiber neuropathy is suspected [2,9]. They help confirm diagnosis, estimate severity, and identify cases in which another neuropathy or focal nerve disorder should be considered. In patients with asymmetry, weakness, rapid progression, motor involvement, or diagnostic uncertainty, they are especially important. The limitation is equally important: conventional nerve conduction studies assess large fibers, not small fibers. A patient with early DPN may therefore have normal electrophysiology despite genuine neuropathic injury [2,13,14]. A normal NCS does not exclude early disease. Recently, point-of-care sural nerve conduction devices have emerged as a pragmatic intermediate option, offering more objective testing than bedside examination and better scalability than full conventional neurophysiology [19-21]. These tools are promising, but they still primarily assess large-fiber function and do not replace specialist evaluation when small-fiber neuropathy is suspected.

Small-Fiber Diagnostic Methods

Small-fiber testing has attracted growing interest because it targets the component of the disease that may appear early. Skin biopsy with intraepidermal nerve fiber density measurement is one of the most established methods [13,14,22]. It provides structural evidence of small-fiber loss and can be helpful in patients with symptoms suggestive of neuropathy but normal nerve conduction studies. The drawbacks are that it is invasive, requires technical expertise, and is not widely available.

Corneal confocal microscopy has emerged as a particularly promising non-invasive method. The cornea contains a dense network of small sensory nerve fibers, and several studies have shown that corneal nerve abnormalities reflect peripheral small-fiber damage in diabetes [23-27]. Corneal confocal microscopy has shown promise for detecting early abnormalities, including in people with impaired glucose tolerance and in patients whose standard bedside tests remain unrevealing [26,27]. It is repeatable and patient-friendly, but access remains limited, and interpretation still requires expertise or validated automated analysis.

Quantitative sensory testing offers a functional approach by measuring thresholds for warm, cold, vibration, and painful stimuli [28]. It can be helpful in early neuropathy, but it is not purely a small-fiber test in every context, because some modalities involve large-fiber function as well. Results also depend on patient attention, understanding, and cooperation, which limits day-to-day reproducibility.

Sudomotor testing adds another angle by assessing autonomic small-fiber function. Devices such as Sudoscan and other sudomotor approaches may detect abnormalities that precede overt loss of protective sensation [29-31]. More specialized autonomic techniques, such as the Quantitative Sudomotor Axon Reflex Test (QSART), can provide additional physiological information, but their wider clinical uptake is constrained by equipment requirements, time, and expertise [30].

Table 1 gives the diagnostic methods for DPN.

**Table 1:** Diagnostic methods for diabetic peripheral neuropathy and their best clinical role MNSI, Michigan Neuropathy Screening Instrument; TCSS, Toronto Clinical Scoring System; UENS, Utah Early Neuropathy Scale; IENFD, intraepidermal nerve fiber density; NCS, nerve conduction studies.

Method	Primary target	Main strength	Main limitation	Best clinical role	Key references
Monofilament/vibration testing	Large-fiber dysfunction and loss of protective sensation	Fast, inexpensive, scalable	Insensitive for early small-fiber disease	Routine annual screening and ulcer-risk assessment	[8,10-12]
Clinical scores (MNSI, TCSS, UENS)	Symptoms plus structured bedside signs	Improves consistency and longitudinal follow-up	Still limited by dependence on clinical abnormalities	Structured bedside assessment and follow-up	[15-18]
Conventional nerve conduction studies	Large fibers	Objective confirmation helps exclude alternative neuropathies	May be normal in early or small-fiber-predominant disease	Diagnostic confirmation and atypical cases	[2,9,13,14]
Point-of-care sural nerve conduction devices	Large-fiber electrophysiology	More objective than bedside testing; easier to deploy than full NCS	Limited phenotyping; not a substitute for specialist testing	Intermediate escalation tool where available	[19-21]
Skin biopsy (IENFD)	Small-fiber structure	Direct evidence of small-fiber loss	Invasive; limited availability	Confirmation of suspected small-fiber neuropathy	[13,14,22]
Corneal confocal microscopy	Small-fiber morphology	Non-invasive and promising for early change	Limited access; analysis expertise needed	Early small-fiber assessment and research/selected clinics	[23-27]
Quantitative sensory testing	Thermal and vibration thresholds	Functional sensory information; non-invasive	Patient-dependent; not purely small-fiber in all contexts	Adjunctive phenotyping	[28]
Sudomotor / autonomic testing	Autonomic small-fiber function	May detect early autonomic involvement	Variable standardization and access	Adjunctive evaluation of suspected small-fiber/autonomic neuropathy	[29-31]
Serum neurofilament light chain	Systemic neuroaxonal injury biomarker	Potential future blood-based adjunct	Investigational and not disease-specific enough yet	Research and future risk stratification	[32,33]
Wearable gait/pressure technologies	Functional gait and plantar-pressure abnormalities	Continuous or real-world monitoring potential	Adjunctive rather than diagnostic	Monitoring, prevention, and digital follow-up	[34,35]

Prediabetes, metabolic neuropathy, and the missed population

A further gap in the standard DPN framework is the patient with neuropathic symptoms, obesity, dyslipidemia, or impaired glucose regulation who may not yet carry a formal diabetes diagnosis. Neuropathy associated with prediabetes and metabolic syndrome is increasingly recognized, and small-fiber involvement may be especially prominent in this setting [4,5,36]. This matters because a narrow focus on established diabetes can delay recognition of metabolically driven neuropathy in patients whose laboratory status or risk factors fall short of overt diabetes. In practice, distal burning pain, thermal sensory disturbance, or autonomic symptoms in a patient with prediabetes, obesity, or metabolic syndrome should not be dismissed simply because monofilament testing and routine NCS are normal.

Emerging and adjunctive approaches (a practical way forward)

Several emerging approaches deserve mention. Serum neurofilament light chain is being studied as a blood biomarker of neuroaxonal injury and has shown associations with diabetic sensorimotor polyneuropathy and peripheral nerve dysfunction, including in recent-onset diabetes [32,33]. At present, however, it should be viewed as investigational rather than a replacement for clinical or physiological assessment. Wearable technologies and digital gait or plantar-pressure tools are also evolving and may improve monitoring, risk prediction, and remote assessment, especially in patients with gait instability or limited access to specialist services [34,35]. Their current role is adjunctive rather than diagnostic, but they may become more useful as validation improves.

Cost, access, and real-world implementation

Cost is an important barrier, but the issue is broader than test price alone. Real-world implementation also depends on equipment availability, trained personnel, referral pathways, test standardization, and whether results change management in a way that improves patient outcomes. Conventional bedside tools remain dominant partly because they are simple and scalable. By contrast, skin biopsy, corneal confocal microscopy, specialized autonomic testing, and even some point-of-care technologies require local infrastructure that many diabetes clinics do not have. Formal cost-effectiveness evidence remains limited for several newer modalities, which makes widespread adoption difficult even when diagnostic performance is promising. A pragmatic model may therefore be tiered access rather than universal deployment: inexpensive screening for all, followed by selective second-line testing in symptomatic, high-risk, or diagnostically uncertain patients.

Practical clinical clues, even when routine testing is normal

Clinicians should look carefully for symptoms and signs that may justify further evaluation, even when routine testing is normal. These include burning feet, painful tingling, thermal sensory disturbance, allodynia, hyperalgesia, nocturnal worsening, reduced sweating, unexplained orthostatic symptoms, early gait insecurity, recurrent footwear trauma, or symptoms that are disproportionate to examination findings. A normal monofilament test should be reassuring for loss of protective sensation, but not for every neuropathy phenotype. In the right clinical context, normal bedside testing should trigger reassessment rather than dismissal.

**Figure 1:**
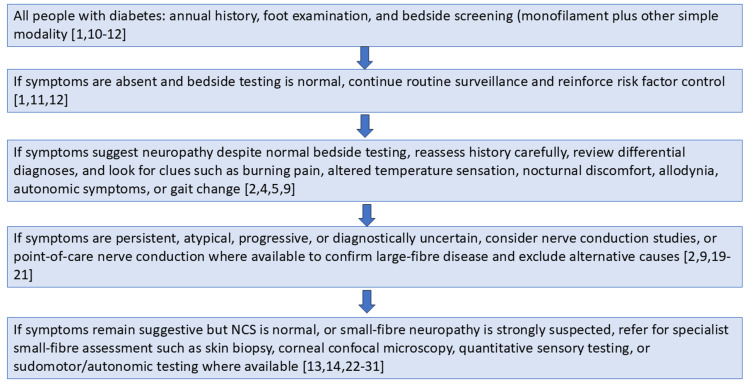
Practical pathway for earlier recognition of diabetic peripheral neuropathy This pathway is based on the literature summarized in this review, including guideline recommendations and studies on conventional, point-of-care, and small-fiber-focused diagnostic approaches [1-2,8-14,19-31].

A practical, risk-based pathway for earlier recognition of diabetic peripheral neuropathy is summarised in Figure 1.

Limitations

This review has several limitations. First, it is a narrative review and therefore does not follow a formal systematic review framework with predefined inclusion criteria, duplicate screening, or quantitative risk-of-bias assessment. Second, diagnostic criteria vary across studies, which limits direct comparison across modalities. Third, access to corneal confocal microscopy, skin biopsy, autonomic testing, and some sudomotor platforms is inconsistent across centers. Fourth, standardization remains incomplete for several promising techniques, particularly in relation to thresholds, operator training, and referral pathways. Fifth, although earlier detection appears biologically and clinically attractive, evidence that every incremental gain in diagnostic sensitivity will translate into better long-term outcomes is still evolving. Finally, newer approaches such as blood biomarkers and wearable technologies remain promising but should currently be regarded as adjunctive rather than established routine standards.

## Conclusions

Traditional bedside tools should not be abandoned. They are practical, inexpensive, and highly relevant for ulcer-risk assessment. However, they should not be mistaken for sensitive tests of early neuropathy. Conventional NCSs remain valuable for confirming large-fiber disease and excluding alternative diagnoses, but a normal result does not rule out early or small-fiber-predominant DPN. The central clinical message is to separate screening, diagnosis, and phenotyping more clearly. In current practice, the most sensible approach is a risk-based, multimodal strategy: universal annual screening for all patients with diabetes, careful attention to neuropathic symptoms, appropriate use of nerve conduction studies for confirmation or atypical presentations, and selective referral for small-fiber testing in symptomatic or high-risk patients when conventional assessment is unrevealing. That is the most realistic way to reduce the number of patients who are diagnosed only after the opportunity for earlier intervention has begun to narrow.
